# Effects of planting density and nitrogen application on the growth of *Xanthium strumarium* subsp. *sibiricum* under manganese stress

**DOI:** 10.3389/fpls.2025.1704841

**Published:** 2026-01-20

**Authors:** Gao Pan, Ling He, Zixin Yu, Meng Chen, Yidan Gao, Zhiliang Li, Wensheng Liu

**Affiliations:** 1 College of Life Science and Technology, Central South University of Forestry and Technology, Changsha, China; 2 Hunan Research Center of Engineering Technology for Utilization of Environmental and Resources Plant, Central South University of Forestry and Technology, Changsha, China

**Keywords:** antioxidant defense, heavy metal tolerance, micronutrient toxicity, nutrient interactions, osmotic regulation, photosynthetic pigments, phytoremediation

## Abstract

**Introduction:**

Planting density and nitrogen (N) application play important roles in plant growth and heavy metal uptake under stress conditions. However, their mechanisms of interaction remain unclear.

**Methods:**

To investigate the effects of planting density and N application on plant growth and manganese (Mn) toxicity alleviation, a pot experiment was conducted to study the variations in growth, physiological and biochemical characteristics, and Mn uptake of *Xanthium strumarium* subsp. *sibiricum* under different Mn concentrations (0.05 and 10 mmol L^-1^), planting densities (3, 42, and 84 individuals m⁻²), and N application rates (0, 5, and 30 mmol L^-1^).

**Results:**

The results showed that with increasing planting density and N application under Mn stress, individual plant biomass first increased and then decreased, while biomass per m^2^ increased. The contents of photosynthetic pigments (chlorophyll a, chlorophyll b, and carotenoids), osmotic adjustment substances (soluble sugars, soluble proteins, and free proline), and antioxidant enzyme activities (SOD, POD, and CAT) also increased initially and then declined. Conversely, the contents of malondialdehyde and relative electrical conductivity decreased. Mn concentrations in the roots, stems, leaves, fruits, and individual plants decreased, whereas total Mn uptake increased with higher planting density and exhibited an initial rise followed by a decline with increasing N application.

**Discussion:**

This study demonstrates that planting density and N applications significantly affect Mn accumulation and the tolerance of *Xanthium strumarium* subsp. *sibiricum* to Mn stress, primarily by promoting the accumulation of osmotic adjustment substances, enhancing antioxidant enzyme activities, and reducing Mn concentration in plant tissues. These findings highlight that appropriate N management and planting density can mitigate Mn toxicity and improve plant resilience under Mn stress.

## 1 Introduction

With the development of industry and economy, soil manganese (Mn) pollution due to human activities, including mining, metallurgy, and chemical industries, has become an important environmental problem (Betancourt et al., 2015; Tang et al., 2019). Mn is an essential micronutrient for plants to maintain normal life activities. However, excess Mn severely inhibits plant growth, development, and physio-biochemical functions and may endanger human health through absorption, enrichment, and transfer into the food chain by plant uptake (Xie et al., 2015; Wang et al., 2022; Hafeez et al., 2022). Mn toxicity is probably the most important growth-limiting factor after aluminum (Al) for plants in acidic soils in some subtropical and tropical areas (Zhao et al., 2017). Therefore, it is urgent to remediate Mn-contaminated soils. Among remediation approaches, phytoremediation is one of the most economical and effective methods, which has broad application prospects (Shah et al., 2020). Moreover, cultivation measures, including planting density and nitrogen (N) application, are often regarded as important management practices in improving plant biomass and heavy metal uptake, thus improving phytoremediation effectiveness (Yan et al., 2017). Although many studies on phytoremediation effects of cultivation measures have conducted at population level under heavy metal stress, few have investigated adaptive responses at the individual plant level (Liu et al., 2009; Lu et al., 2010; Zheng et al., 2023). However, individual plant adaptive traits are solid foundations for plant population, which play key roles in clarifying the phytoextraction mechanisms at population level. Therefore, understanding how planting density and N application affect individual plant growth and Mn stress adaptation is critical for improving phytoremediation efficiency.

Planting density, which significantly affects photosynthetic efficiency and nutrient absorption, is a key factor influencing plant growth and heavy metal uptake (Jacobs et al., 2018; Zhang and Tielboerger, 2020; Zou et al., 2021; Zheng et al., 2023). Most studies investigated the effects of planting density under contaminated conditions on phytoremediation at population level, which showed that population biomass initially proportionally increased, leveled off, and then remained constant with rising planting density, and proper plant density could improve phytoextraction efficiency (Liu et al., 2009; Lu et al., 2010; Li et al., 2016; Zhu et al., 2020; Viana et al., 2021; Zou et al., 2021; Zheng et al., 2023). At individual plant level, planting density greatly influences the competition for light, water, and nutrient among individual plants, which would affect plant growth (Zhu et al., 2020). Moreover, plants would share and compete not only for light, water, and nutrients but also for trace metals under stress (Wang et al., 2020; Zheng et al., 2023). Thus, individual plant biomass and heavy metal absorption would decrease under high planting density due to competition (Hansi et al., 2014). This hypothesis has so far been tested by many studies. For example, Liu et al. (2009) showed that high planting density could decrease plant growth and absorption of cadmium (Cd) and zinc (Zn) for *Sedum plumbizincicola*. Lu et al. (2010) found that high planting density could decrease individual plant absorption of plumbum (Pb) and arsenic (As) for *Boehmeria nivea*. However, it is also reported by Jacobs et al. (2018) that concentrations of Cd and Zn in the plant of *Noccaea cadeulescens* increased with enhancing planting density and stress, because increased soil exploration due to the metal-foraging behavior of plants through enhanced root proliferation might increase the metal uptake of closely interacting individuals under high planting density. This showed that the effect and mechanism of planting density on growth and metal uptake remained unclear.

N is one of the most important nutrients and a major factor influencing plant growth and metal uptake under stress (Jalloh et al., 2009; Jacobs et al., 2018). Both N deficiency and excess significantly reduce plant production, and optimal N application can increase plant yield (Hu et al., 2025). It was reported that heavy metals may cause essential nutrient deficiency and even change the concentrations of basic nutrients such as N in plant tissues (Panković et al., 2000). N application can promote plant growth and alleviate the toxic effect of heavy metals in real soil conditions by increasing the amounts of stromal proteins, photosynthetic capacity of leaves, and the plant growth (Panković et al., 2000; Jalloh et al., 2009; Hou et al., 2024; Souza Junior and Monteiro, 2024; Chen et al., 2025). Thus, to reveal how heavy metal toxicity may be reversed by elevated macronutrient supply is needed. Moreover, N application can lower soil pH, increase metal solubility, and thereby enhance plant uptake. For example, Dong et al. (2017) found that N supplementation can alleviate Cd stress and increase the enrich ability for *Trifolium repens*. Conversely, it was also reported that N treatments resulted in lower Cd and/or Zn shoot concentrations in the experiments, because N might compete with heavy metal for root absorption (Jacobs et al., 2018). This illustrated that the effect and mechanism of N application on plant growth and heavy metal uptake under stress remain unclear. Additionally, as the most important plant management practices to improve phytoremediation effectiveness, planting density and N application would influence each other (Yan et al., 2017; Hu et al., 2025). The combination of N fertilizer and planting density had great effects on plant production and metal uptake (Jacobs et al., 2018). However, there has been little exploration into the effects of N levels and planting density coordinate to regulate plant growth under heavy metal stress.

Excess Mn can induce high synthesis of reactive oxygen species (ROS) in plant cells and cause oxidative stress for plants (Souza Junior and Monteiro, 2024). A key protective mechanism involves antioxidant enzymes such as superoxide dismutase (SOD), peroxidase (POD), and catalase (CAT), which mitigate oxidative damage (Jalloh et al., 2009). Moreover, Mn toxicity can enhance lipid peroxidation in plant cells, which is reflected by increased malondialdehyde (MDA) content. Proline is also an important amino acid in plants under stress that prevents oxidation of cells from inside (Pan et al., 2019a). Therefore, the variations of physiological and biochemical characteristics are important indicators of plant damage and alleviation mechanisms of Mn stress (Lei et al., 2007; Pan et al., 2019b).

*Xanthium strumarium* Linnaeus subsp. *sibiricum* (Patrin ex Widder) Greuter, an annual herb of the Asteraceae family, is widely distributed across China and characterized by rapid growth and high individual biomass production. Moreover, this plant can survive in various habitats. It was reported that this plant had great tolerance to Mn and could accumulate a high amount of Mn, and it could grow and set flowers and fruits normally in manganese mine wasteland (Pan et al., 2019b). Therefore, it is an important candidate plant for Mn phytoremediation (Pan et al., 2019a). Field surveys also revealed that this plant could form a single-dominant community with different plant densities in manganese mine wasteland. This suggested that this plant is a suitable material for investigating the effects of planting density on growth under Mn stress.

In this study, a pot experiment was carried out to investigate the interactive effects of planting density, N application, and Mn stress on plant growth, physiological and biochemical characteristics, and Mn accumulation characteristics of *X. strumarium* subsp*. sibiricum*. We hypothesized that the densification of *X. strumarium* subsp*. sibiricum* planting with proper N addition in a Mn-polluted soil to an optimized level would increase plant biomass production, thereby resulting in greater accumulation of Mn. We also hypothesized that *X. strumarium* subsp*. sibiricum* could improve heavy metal tolerance by adjusting physiological status under Mn stress with different planting density and N application. This study aimed to determine (1) how planting density and N application affect growth under Mn stress; (2) whether Mn uptake increases with higher density and N application; and (3) the physiological mechanisms underlying plant adaptation to Mn toxicity.

## 2 Materials and methods

### 2.1 Seed collection

Seeds of *X. strumarium* subsp*. sibiricum* were collected in Xiangtan manganese tailings, Hunan Province, China, in November 2022 (28°03′E, 112°55′N). Field investigations showed that the soil Mn concentration was 52,319.25 ± 2,507.16 mg kg^−1^. *X. strumarium* subsp*. sibiricum* covered approximately 650 m^2^ in area, and the plant density was approximately 44 individuals m^−2^, which ranged from 34 individuals m^−2^ to 85 individuals m^−2^. This plant was approximately 0.5 m in height and set fruits normally. Over 100 well-growing plants of *X. strumarium* subsp*. sibiricum* were selected, and the minimum distance between each other was over 2.0 m. Then, the mature and plump fruits were sampled and brought back to the laboratory by cloth bags. The seeds were stored at room temperature until the beginning of seed germination.

### 2.2 Pot experiment

Seedling preparation were conducted in the greenhouse of Central Southern University of Forestry and Technology in March 2023. The air temperature and relative humidity of the greenhouse ranged 20°C-30°C and 45%-85%, respectively. The seeds were sown and germinated in plastic pots (71 cm in length, 45.5 cm in width, and 18 cm in height) filled with sand.

After 40 days, the seedlings were at the five-leaf stage, and then the plants were selected uniformly and transplanted into the pots (71 cm in length, 45.5 cm in width, and 18 cm in height, containing 30 kg of substrate). The pot experiment was carried out in a completely randomized design in the same greenhouse used for seed germination and seedling cultivation. The experiment consisted of two levels of Mn concentrations (with 0.05 *vs*. 10 mmol/L Mn^2+^ concentration; expressed as no Mn stress and Mn stress hereafter), three levels of planting densities (3, 42, and 84 individuals m^−^²; expressed as low planting density, middle planting density, and high planting density hereafter, respectively), and three levels of nitrogen application (0, 5, and 30 mmol L^−1^; expressed as low N application, middle N application, and high N application hereafter, respectively). Therefore, there were 2 Mn concentrations × 3 planting densities × 3 nitrogen applications × 8 replicates = 144 pots in total.

The substrate used was a 1: 1 (v/v) mixture of sand and perlite. The sand used for this mixture was collected from the campus of Central South University of Forestry and Technology. The sand was rinsed, air-dried, and then filtered with a 2-mm sieve to remove grit and facilitate subsequence manipulations. Mn concentrations, N applications, and planting densities were set according to Pan et al. (2019a, b), Ye et al. (2021), and Jacobs et al. (2018), respectively. Moreover, the field plot survey in Xiangtan manganese tailings suggested that the average plant density was approximately 44 individuals m^−2^, which showed that the middle planting density of 42 individuals m^−2^ is appropriate. Planting densities were set by transferring 1, 14, and 28 individual seedlings of *X. strumarium* subsp*. sibiricum* to each pots, which refers to 3, 42, and 84 individuals m^−^², respectively. To simulate Mn-contaminated substrate and N application, Mn was added to each pot in the form of MnCl_2_, and N fertilization was applied as CO(NH_2_)_2_ (urea). The substrate was then watered and stirred every day to make sure that Mn and N were distributed evenly in the substrate. Prior to Mn stress and N applied treatment, the transferred seedlings were cultivated in the greenhouse for 1 week of adaptation. Fertilizers and Mn stress were applied with three splits, 40%, 30%, and 30% every 7 days. Pots were watered with Hoagland’s nutrient solution as necessary. The Hoagland’s nutrient solution contained (in mg/L) 607 K_2_SO_4_, 115 NH_4_H_2_PO_4_, 493 MgSO_4_, 20 C_10_H_12_FeN_2_NaO_8_·3H_2_O, 15 FeSO_4_·7H_2_O, 2.86H_3_BO_3_, 4.5 Na_2_B_4_O_7_·10H_2_O, 2.13 MnSO_4_, 0.05 CuSO_4_, 0.22 ZnSO_4_, and 0.02 (NH4)_2_SO_4_. Solution pH was adjusted to 5.5 ± 0.2 with NaOH as required. All the pots were watered every day, and the amount of water applied was determined by weighing a few pots to estimate moisture loss and adding enough water to keep around 60% soil water holding capacity. In addition, to minimize the position effects, pots were repositioned every 7 days.

### 2.3 Morphological measurements

After 90 days, three individual plants from each treatment (one from each pot) were sampled prior to harvest. Plant height (cm) was measured as the soil surface to the top of the plant by using a meter ruler. The stem diameter (mm) of the first true leaf was measured by using a digital vernier caliper (ST22302, SG Tools, Hangzhou, China).

Plant biomass was measured following plant harvested, and the individual plant was divided into four parts (root, stem, leaf, and fruit) and washed by deionized water, respectively. All plant materials were kept and dried for 2 h at 105°C, followed by 48 h at 70°C to achieve a constant weight. The biomass of roots, stems, leaves, and fruits of individual plant were determined by using an electronic balance with 0.0001 accuracy. The population biomass was estimated as the dry biomass per pot divided by the area of the pot.

### 2.4 Determination of physiological and biochemical characteristics

In order to assess the following parameters, the remaining plants in each pot, i.e., five plants in each treatment, were sampled. Chlorophyll content was estimated following a previously described method (Shah et al., 2020). Fresh leaves (100 mg) were treated with acetone in 80% acetone, centrifuged (4,000 g, 10 min, 4°C), and then filtered. The concentrations of chlorophyll a, chlorophyll b, and carotenoid were computed after the sample solutions were read using a spectrophotometer at wavelengths of 663, 645, and 470 nm on a spectrophotometer (SPARK 10 M).

A leaf sample of approximately 0.2 g was ground in a mortar with liquid nitrogen by using sodium phosphate buffer (pH 7.2). The homogenate was centrifuged, and the supernatant solution was collected to determine enzyme activity. Activity of SOD was measured using the nitro blue tetrazolium (NBT) method (Han et al., 2015). The absorbance was measured at 560 nm. POD and CAT were determined as the methods of Gao (2000). POD activity was expressed as the variation of absorbance per minute. The reaction was measured at wavelength of 470 nm. CAT activity was recorded at wavelength of 240 nm.

The amounts of soluble sugar content and free proline content were determined as the methods of Gao (2000) (with slight modification). Free proline was estimated in the powdered dry leaves while homogenized in 3% sulfosalicylic acid according to Bates et al. (1973) using ninhydrin reagent. The concentration of proline was estimated using a standard curve obtained after plotting the absorbance of pre-prepared proline concentration stocks (20, 40, 60, 80, and 100 ppm) and was expressed in μmol g^−1^. The absorbance was determined at a wavelength of 520 nm using a spectrophotometer. Soluble protein content was determined using the method of Bradford (1976) with bovine serum albumin (BSA) as a standard. Leaf samples were homogenized in sodium phosphate buffer (pH 7.0) and centrifuged. 1 mL of Bradford solution was added to 100 mL crude extract. The absorbance was recorded at a wavelength of 595 nm for estimation of total protein contents. The protein concentration was calculated from a BSA standard curve.

MDA content was determined using the thiobarbituric acid (TBA) method as described by Heath and Packer (1968). 5 mL of trichloroacetic acid (TCA; 0.1% w/v) was used to extract 0.2 g of fresh leaf. The mixture was then centrifuged at 12,000 g for 5 min. Thiobarbutaric acid (TBA; 4 mL, 0.5%) was used to treat the sample solution with TCA (20%). In a water bath, the mixture was heated at 90°C for 30 min. The supernatant was collected, and the absorbance was recorded at wavelengths of 532 and 600 nm. Three replicates of each treatment were used, and each replication was the average of five readings.

### 2.5 Mn concentration analysis

Mn concentration was determined according to the methods of Xiao et al. (2020). The dried tissues of different plant parts (roots, stems, leaves, fruits) were ground into fine powder and sieved through a 1-mm nylon sieve. 0.5 g sample powder was digested with a solution containing 4 mL of HNO_3_ (71%, w/w) and 1 mL of HCl (32%, w/w) and placed in a muffle furnace at 300°C for 2 h followed by 5 h at 500°C on a hot plate. Then, the digested sample was made up to 25 mL and analyzed by a Perkin Elmer Analyst 800 AAS at a wavelength of 228.8 nm. Triplicate samples and blanks were taken as the quality controls during the analysis process. Total individual plant Mn concentration was determined by calculating the dry biomass and Mn concentration of each organ (mg kg^−1^) (You et al., 2022). Bioconcentration factor (BCF), translocation factor (TF), and metal uptake were used to evaluate plants phytoremediation efficiency (Nie et al., 2016). Mn uptake of roots, stems, leaves, fruits, individual plant, and the whole population were calculated by multiplying the Mn concentration and the dry biomass together (Viana et al., 2021). All of the measurements were performed in three replicates.

### 2.6 Statistical analysis

Three-way ANOVA was applied to examine the effects of Mn stress, planting density, N application, the interaction effect of three factors and their impacts on the growth (i.e., root biomass, stem biomass, leaf biomass, fruit biomass, total biomass, and root–shoot ratio), Mn levels (i.e., Mn concentration in root, stem, leaf, fruit, total individual plant, and Mn uptake in shoots, roots, and the whole population), and physiological and biochemical characteristics. All data were checked for normality using the Kolmogorov–Smirnov test and for homogeneity of variance using Levene’s test. Data were log-transformed if necessary to ensure assumptions of normality and homogeneity of variances. The mean values of each treatment were subjected to multiple comparisons by the least significant difference (LSD) test (p < 0.05). All statistical analyses were performed by using SPSS software version 19.0 (IBM Corp., Armonk, NY, USA). Graphs were generated using OriginPro 8.5. Data are presented in tables and figures as the means ± standard deviations (n = 5).

## 3 Results

### 3.1 Plant biomass

**Figure 1:**
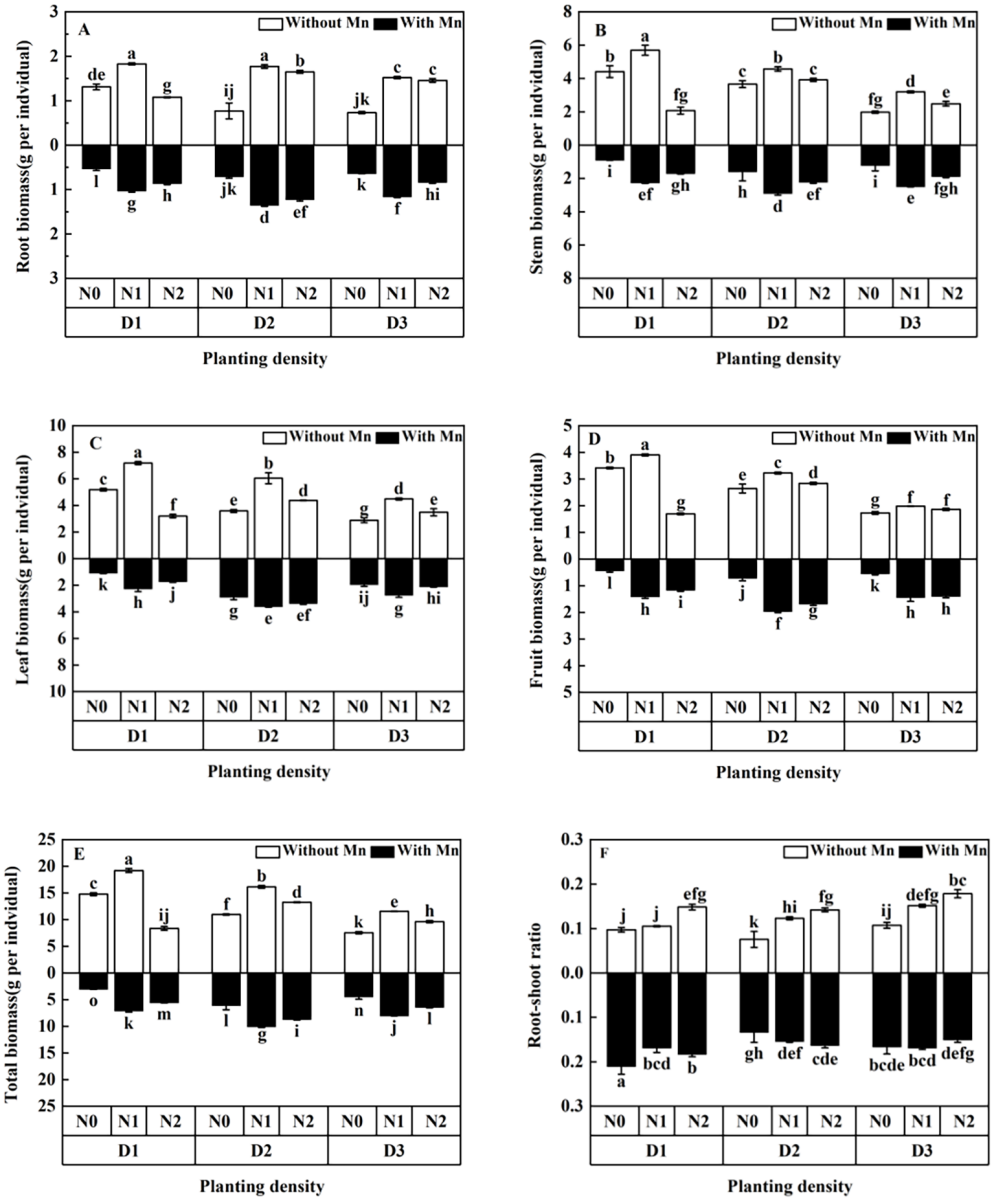
Effects of planting density and nitrogen application on the root biomass **(A)**, stem biomass **(B)**, leaf biomass **(C)**, fruit biomass **(D)**, total biomass **(E)**, and root–shoot ratio **(F)** of *Xanthium strumarium* subsp*. sibiricum* under Mn stress. Different lowercase letters represent significant differences among treatments (*P* < 0.05).

Three-way ANOVA showed significant interaction effects of Mn stress, planting density, and N application on biomass components (**Supplementary Table S1**). It showed that Mn stress significantly reduced total and organ-specific biomass (**Figures 1A-E**; *p* < 0.01). Biomass exhibited an increasing response, peaking at middle planting density (42 individuals m^−^²) and N level (5 mmol L^−1^) under Mn stress (**Figure 1**). Middle planting density increased total biomass by 36%-99% relative to the other densities. Meanwhile, the root–shoot ratio decreased with the rising planting density and N application under Mn stress (**Figure 1F**, **Supplementary Table S1**).

### 3.2 Physiological and biochemical characteristics

Three-way ANOVAs with Mn stress, planting density, and N application and their interaction are presented in **Supplementary Tables S2**-**S4**, and **S5**. Most of the analyzed physiological and biochemical variables were highly affected by each of the three factors separately and their interactions.

**Figure 2:**
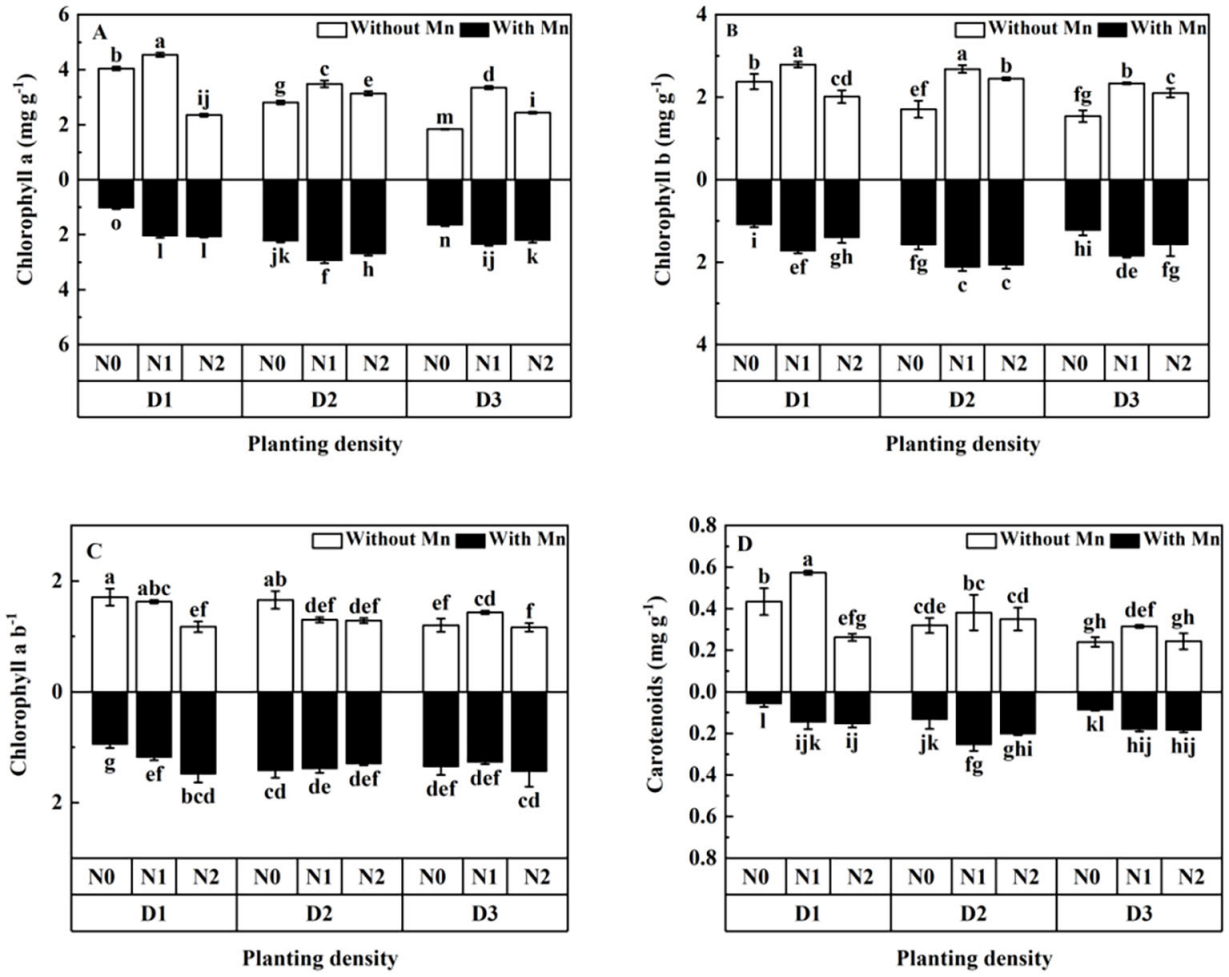
Effects of planting density and nitrogen application on the contents of chlorophyll a **(A)**, chlorophyll b **(B)**, chlorophyll a/b **(C)**, and carotenoids **(D)** of *X. strumarium* subsp*. sibiricum* under Mn stress. Different lowercase letters represent significant differences among treatments (*P* < 0.05).

Mn stress decreased the contents of photosynthetic pigment (chlorophyll a, chlorophyll b, and carotenoids) (**Figure 2**; *p* < 0.05). Both planting density and N application markedly influenced photosynthetic pigment, and the contents of photosynthetic pigment first increased to the peak value and then decreased with the rising planting density and N application under Mn stress (**Figures 2A–D**). Plants in the treatment of middle planting density and middle N application had the highest contents of chlorophyll a, chlorophyll b, and carotenoids under stress (**Figure 2**).

**Figure 3:**
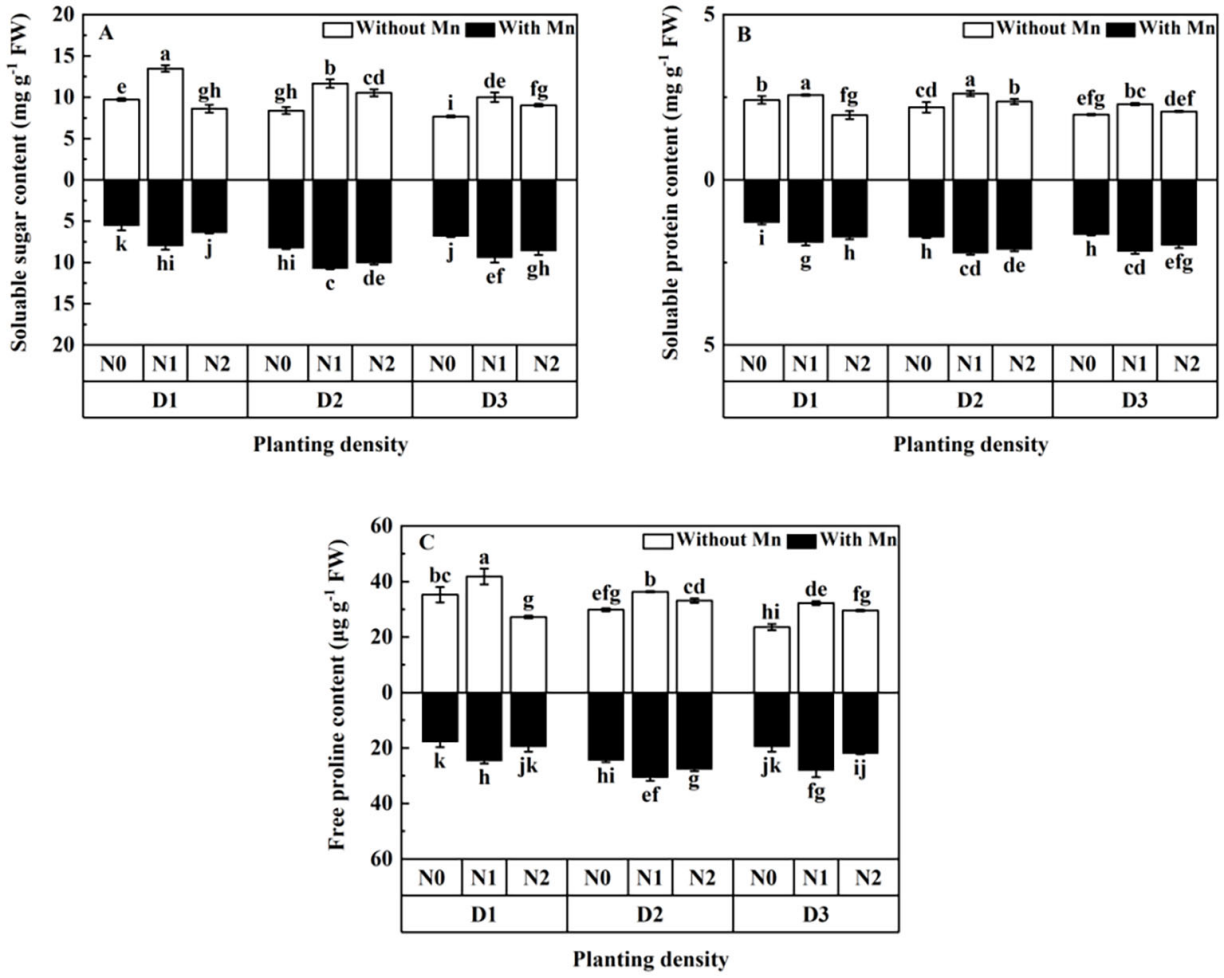
Effects of planting density and nitrogen application on the contents of soluble sugar **(A)**, soluble protein **(B)**, and free proline **(C)** of *X. strumarium* subsp*. sibiricum* under Mn stress. Different lowercase letters represent significant differences among treatments (*P* < 0.05).

The values of soluble sugar, soluble protein, and free proline (osmotic regulation substances) of the plants without Mn were significantly higher than that with Mn (**Figure 3**; *p* < 0.01). Osmotic regulation substances increased firstly and then decreased with the increasing planting density and N application (**Figure 3**). Plants in the treatment of middle planting density and middle N application had the highest contents of osmotic regulation substances under Mn stress (**Figure 3**).

**Figure 4:**
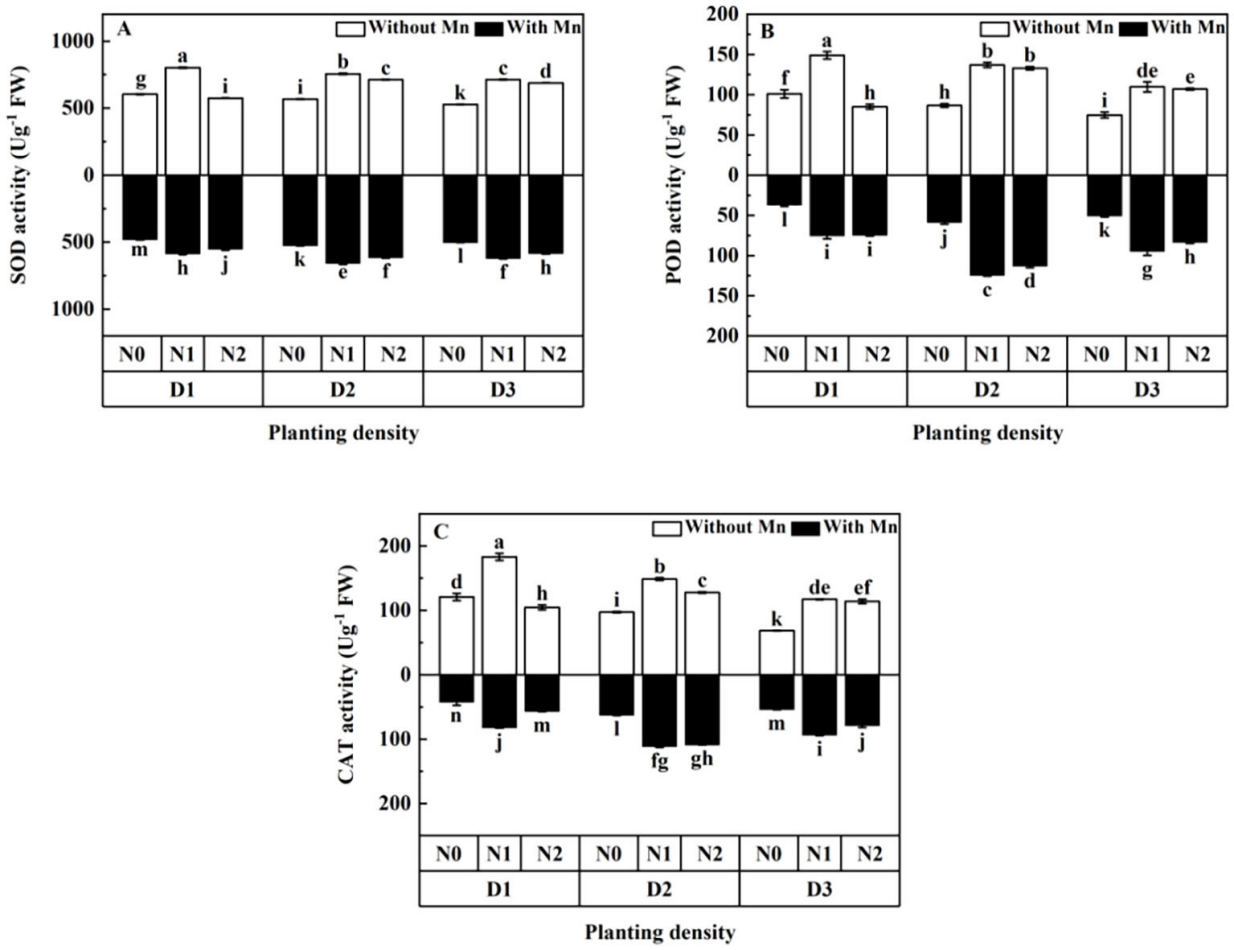
Effects of planting density and nitrogen application on the activities of superoxide dismutase (SOD) **(A)**, peroxidase (POD) **(B)**, and catalase (CAT) **(C)** of *X. strumarium* subsp*. sibiricum* under Mn stress. Different lowercase letters represent significant differences among treatments (*P* < 0.05).

The activities of SOD, POD, and CAT (antioxidant enzymes) of plants without Mn application were higher than that with Mn stress (**Figure 4**; *p* < 0.01). Antioxidant enzymes first increased to the peak value and then dropped with the increasing planting density and N application under Mn stress (**Figure 4**). The plants with middle planting density and middle N application had the highest activity of SOD, POD, and CAT under Mn stress.

**Figure 5:**
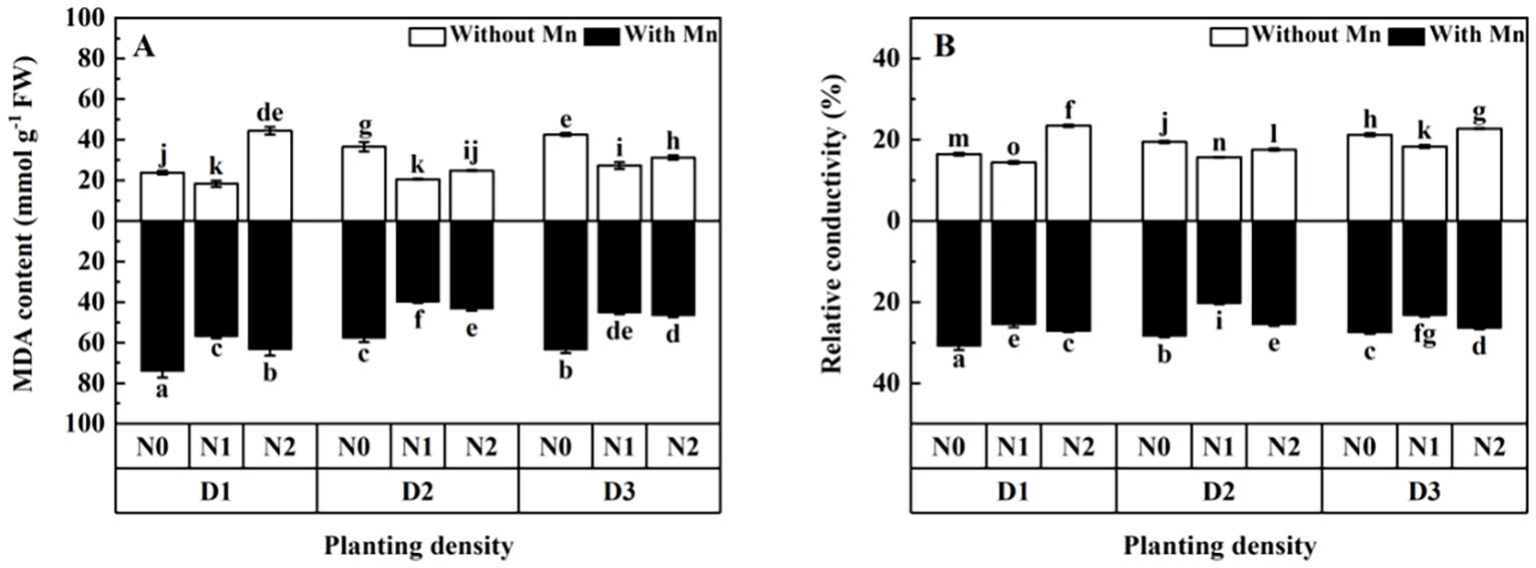
Effects of planting density and nitrogen application on the malondialdehyde (MDA) content **(A)** and relative conductivity **(B)** of *X. strumarium* subsp*. sibiricum* under Mn stress. Different lowercase letters represent significant differences among treatments (*P* < 0.05).

MDA content and relative conductivity of plants with no Mn application were higher than that with Mn stress (**Figure 5**; *p* < 0.01). MDA content and relative conductivity decreased firstly and then increased with the increasing planting density and N application (**Figure 5**). Plants with middle planting density and N application had the lowest values of MDA and relative conductivity than other treatments under Mn stress.

### 3.3 Mn richness

Three-way ANOVA analysis showed the significant difference between planting density, N application, and Mn stress on the Mn concentration of *X. strumarium* subsp*. sibiricum* (**Supplementary Tables S6**-**S8**).

**Figure 6:**
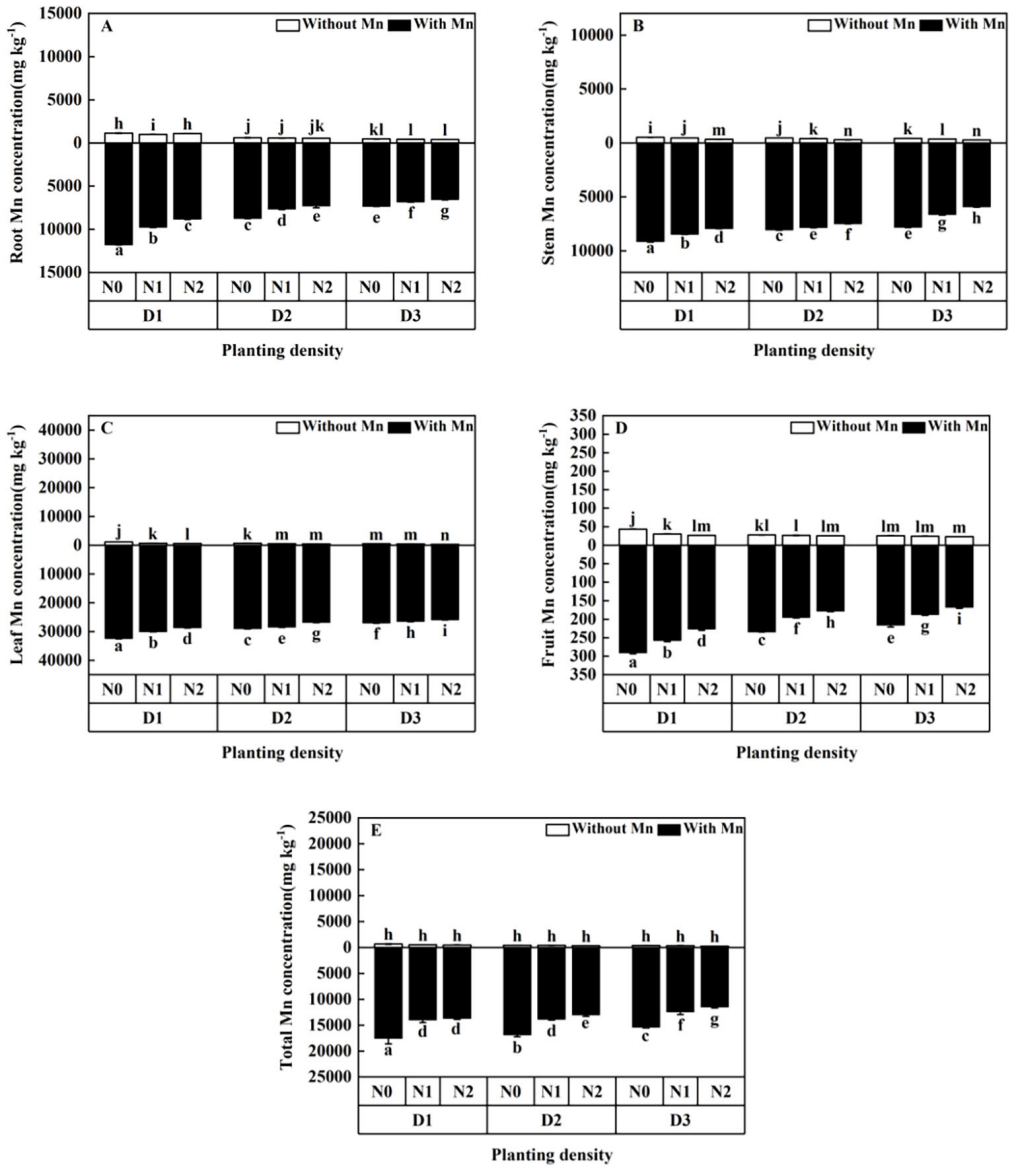
Effects of planting density and nitrogen application on the root Mn accumulation **(A)**, stem Mn accumulation **(B)**, leaf Mn accumulation **(C)**, fruit Mn accumulation **(D)**, and total Mn accumulation **(E)** of *X. strumarium* subsp*. sibiricum* under Mn stress. Different lowercase letters represent significant differences among treatments (*P* < 0.05).

Mn concentrations in the plants without Mn application were significantly lower than that with Mn application (**Figure 6**; *p* < 0.01). Mn concentrations in different parts of the plant decreased with the increasing planting density and N application under Mn stress (**Figure 6**). The plants with high planting density (84-individuals m^−2^) and high N application (30 mmol L^−1^) had the lowest Mn concentrations under Mn stress, which decreased by 25.16% in roots (**Figure 6A**), 13.06% in stems (**Figure 6B**), 11.55% in leaves (**Figure 6C**), 22.06% in fruits (**Figure 6D**), and 21.91% in total individual plants (**Figure 6E**) compared with plants with low planting density (three individuals/m²) and N application (without N application).

**Figure 7:**
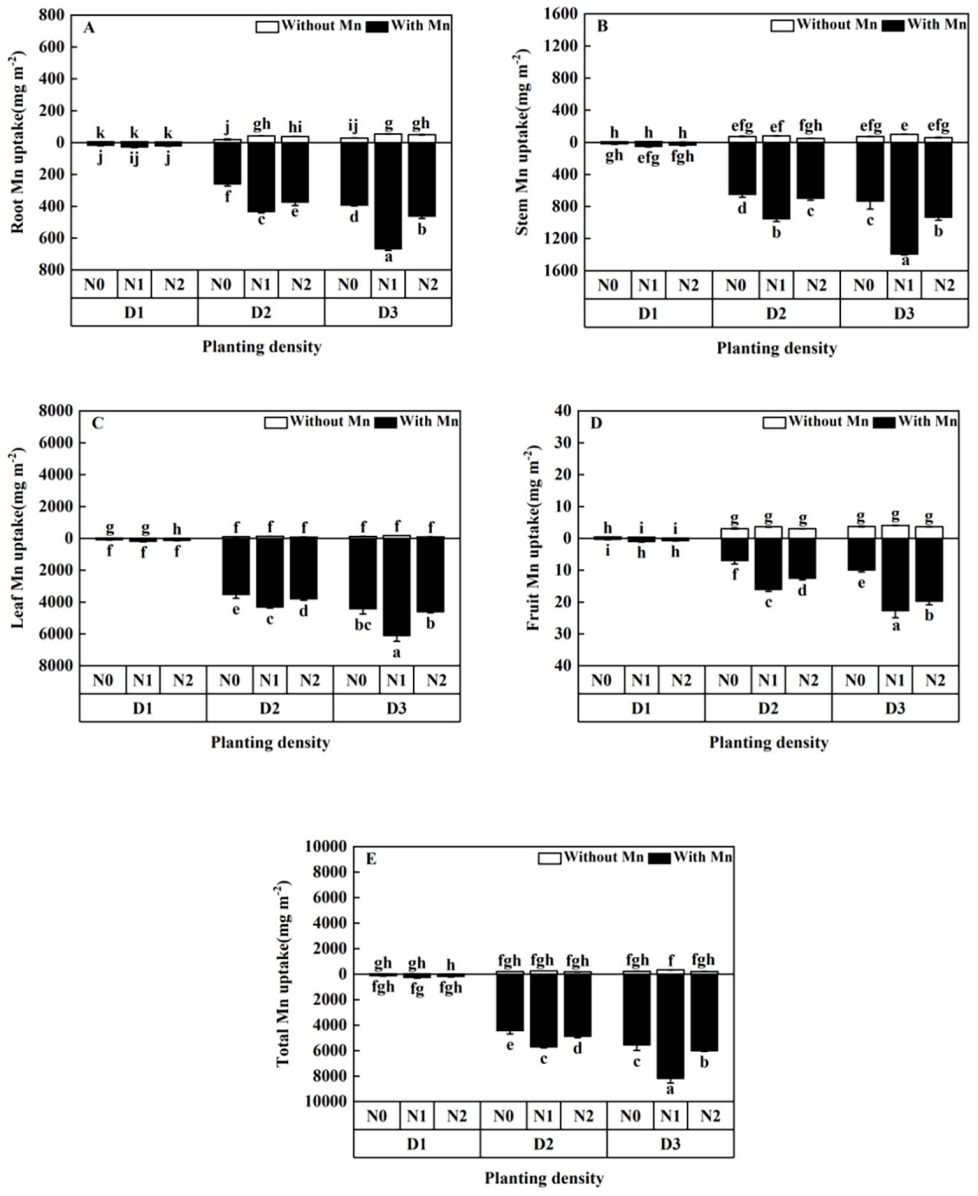
Effects of planting density and nitrogen application on the root Mn enrichment **(A)**, stem Mn enrichment **(B)**, leaf Mn enrichment **(C)**, fruit Mn enrichment **(D)**, and total Mn enrichment **(E)** of *X. strumarium* subsp*. sibiricum* under Mn stress. Different lowercase letters represent significant differences among treatments (*P* < 0.05).

Mn uptake of the plants without Mn treatment was significantly lower than that with Mn (**Figure 7**; *p* < 0.01). Mn uptake of different parts of the plant increased with the increasing planting density and increased firstly and then decreased with the rising N application (**Figure 7**). Plants with high planting density (84 individuals m^-^²) and middle N application (5 mmol L^−1^) had the highest Mn concentration under Mn stress, which increased to 35.29 times in roots (**Figure 7A**), 56.21 times in stems (**Figure 7B**), 57.38 times in leaves (**Figure 7C**), 59.41 times in fruits (**Figure 7D**), and 54.42 times in total individual plants (**Figure 7E**), compared with plants with low planting density (three individuals m^−^²) and low N application (without N application).

**Figure 8:**
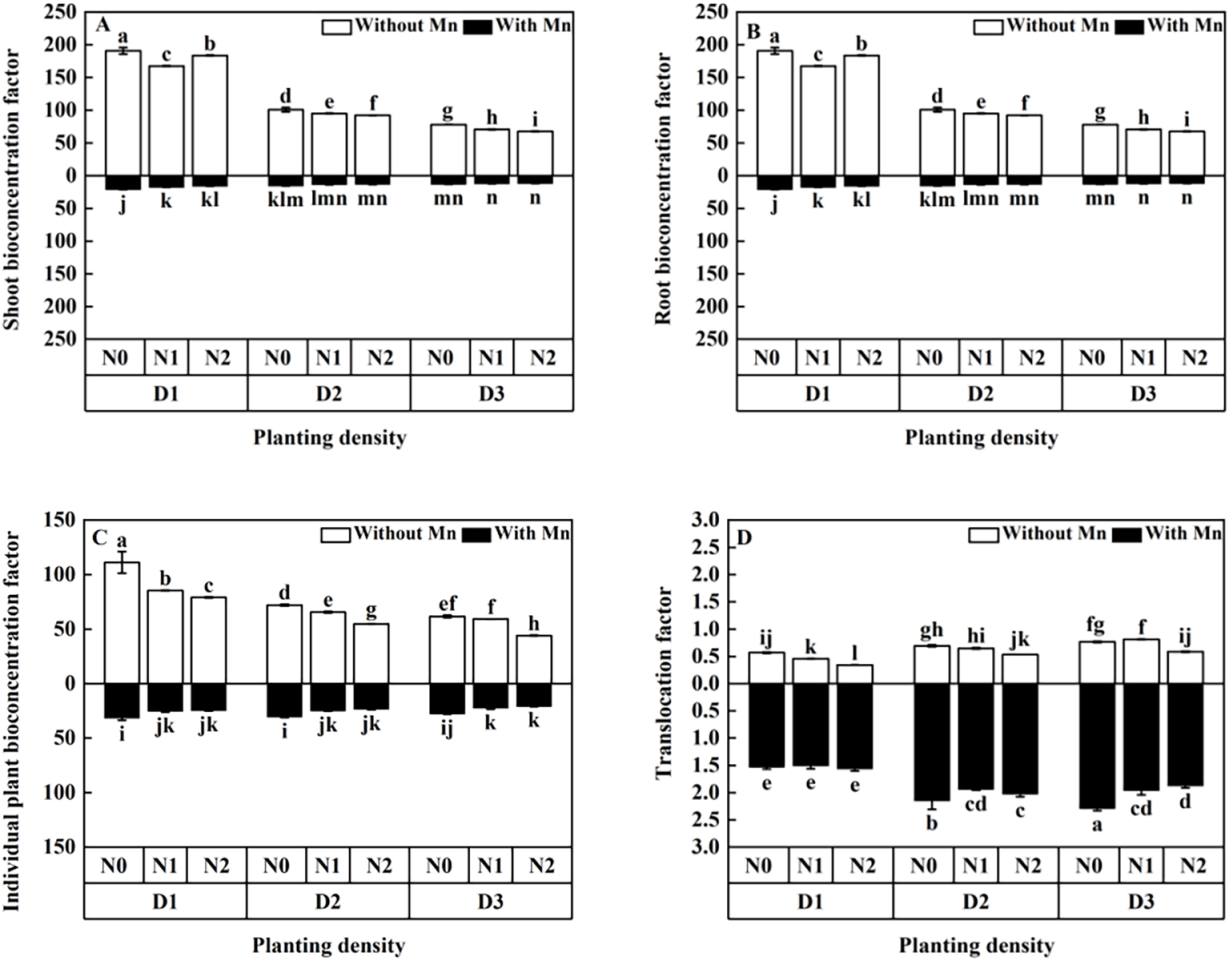
Effects of planting density and nitrogen application on the aerial part bioconcentration factor **(A)**, root bioconcentration factor **(B)**, bioconcentration factor **(C)**, and translocation factor **(D)** of *X. strumarium* subsp*. sibiricum* under Mn stress. Different lowercase letters represent significant differences among treatments (*P* < 0.05).

BC (bioconcentration factor) of shoots, roots, and individual plant decreased with the increasing planting density and N application (**Figures 8A-C**). Translocation factor increased with the increasing planting density, whereas it decreased with the rising N application under Mn stress (**Figure 8D**).

## 4 Discussion

### 4.1 The effects on plant biomass under Mn stress

Plant biomass variation is one of the most important indicators determining plant tolerance to stress (Souza Junior and Monteiro, 2024). The present study showed that individual plant biomass of *X. strumarium* subsp*. sibiricum* increased first and declined afterward with the rising planting density under stress (**Figure 1**). This is consistent with the results of Jacobs et al. (2018) and Wang et al. (2023a) under heavy metal stress. This pattern is due to intraspecific competition for resources and dilution effect caused by planting density under stress. It is known that plants share and “compete” not only for resources but also for heavy metal toxins, which is called “dilution effect” (Hansi et al., 2014). As planting density increases under stress, more individual plants would produce higher productivity with sufficient light, water, and nutrient as planting density increased. Meanwhile, Mn toxin dilution by plants in denser plant stands further promotes plant growth and causes more biomass (Hansi et al., 2014). However, when planting density increases to a certain high level, extra high planting density (84 individuals m^−2^) is likely to cause strong intraspecific competition for light, nutrients, and water in a certain spatial range, and limited resources would result in low biomass production. Therefore, the competition among individual plants caused by high planting density plays key roles in plant biomass under Mn stress, and the planting density of 42-individuals m^−2^ may be an appropriate density for plant growth.

This study illustrated that N application under Mn stress showed the effect of “low-promotion, high-inhibition” on individual plant biomass (**Figure 1**). This is consistent with that of He et al. (2022) and Wang et al. (2023b). The reason is that excessive heavy metals would inhibit plant growth and cause essential nutrient deficiency including basic nutrients such as N in plant tissues. As a main nutrient for plant growth, N supply may alleviate the toxic effect of Mn by increasing the amounts of stromal proteins and photosynthetic capacity of leaves, thus enhancing plant growth (Panković et al., 2000). Moreover, proper N application would supply enough nutrients for plant and increase plant growth. However, too high N application would destroy the nutrient balance and decrease the absorb of other essential nutrients, resulting in low plant production (Wang et al., 2023b).

The present study revealed that the root–shoot ratio of *X. strumarium* subsp*. sibiricum* decreased with the rising planting density and N application under Mn stress (**Figure 1**). This is in agreement with the results obtained from Wang et al. (2023a). It indicates that seriously competition for resources due to rising planting density and N application may occur. For example, light may become a limiting factor as planting density and N addition increased to a certain high level. To survive under this environment, plants would improve the investment in shoots to acquire more light. Alternatively, less root biomass at high planting density would decrease Mn uptake and enhancing plant growth under stress. Therefore, a low root–shoot ratio may be an important mechanism for plants to adapt to heavy metal stress.

### 4.2 The effects on physiological and biochemical characteristics

Chlorophyll a, b and carotenoids are all essential elements for photosynthesis, which are important indicators of plant photosynthesis ability. They are very sensitive to heavy metal stresses (Xiao et al., 2020). Under heavy metal stress, the structure of leaf chloroplast might be damaged, and the contents of leaf chlorophyll would decrease. Thus, leaf chlorophyll contents can provide valuable information on photosynthesis intensity, physiological status of plants, and damaged status of plants (Jalloh et al., 2009). The present study showed that leaf chlorophyll of *X. strumarium* subsp*. sibiricum* first increased to the peak value and then dropped with the rising planting density and N application under Mn stress (**Figure 2**). Zeng et al. (2015) revealed that high planting density decreased chlorophyll content under stress. Chen et al. (2025) and Dong et al. (2017) revealed that N supplementation increased chlorophyll content under heavy metal stress. The present result is consistent with Zeng et al. (2015); Dong et al. (2017), and Chen et al. (2025). This illustrated that planting density and N application had great impacts on chloroplast content under stress. The reason lies in that proper planting density and N addition would reduce the damage of chlorophyll by Mn stress through dilution effect with enhancing biomass, and plants would increase chlorophyll contents to improve the photosynthetic capacity (Wang et al., 2017; Souza Junior and Monteiro, 2024). However, when the planting density and N supplying increased to a certain level, serious intraspecific competition for resources between individual plants would hurt plants due to the lack of light and essential nutrients, and chloroplasts would be severely damaged and could not produce more chlorophyll. Thus, chlorophyll would break down, and the content of chlorophyll would decrease. This result is in line with that of individual plant biomass, which illustrates that plant biomass increased firstly and then decreased with the rising planting density and N application under Mn stress (**Figure 1**).

Soluble sugar, soluble protein, and free proline are all osmotic regulation substances. They can regularize osmotic pressure of plants under stress for absorbing more water and maintain proper water balance destroyed by Mn stress, even under low stomatal conductance situations (Huang et al., 2019). The present study illustrated that osmotic regulation substances increased firstly and then decreased with the rising planting density and N addition under Mn stress. It was reported that osmotic regulation substances increased with the rising planting density (for *C. camphora*; Wang et al., 2017) and N addition (for *Citrullus lanatus*; Wang et al., 2023b). The present study was in agreement with the results obtained from Wang et al. (2017) and Wang et al. (2023b). This is due to dilution effect with enhancing biomass by proper planting density and N addition (Wang et al., 2023b). In addition, N application can enhance the amounts of stromal proteins and biosynthesis of soluble sugar, soluble protein, and P5CS, and then the contents of osmotic regulation substances would increase (Hou et al., 2024). However, as planting density and N addition attained to a certain level, the cell structure of the leaves would be damaged due to the shortage of light and essential nutrients for plants and would hinder the synthesis of protein, sugar, and proline. As the decomposition rate of existing protein and sugar accelerated, it would result in the reduction in biochemical and physiological activities of plant cells (Mu et al., 2022). It is worth noting that plants can increase the water uptake by maintaining the osmotic potential thereby reducing the impacts of stress (Wang et al., 2023b). This is an important mechanism for plants to adapt to Mn stress.

Mn stress would break the metabolic balance of reactive oxygen species (ROS) and cause oxidative damage, resulting in oxidative stress (Mittler, 2002). Thus, oxidative stress plays a central role in heavy metal toxicity. To deal with oxidative damage, antioxidant enzymes such as SOD, POD, and CAT would be activated (Wang et al., 2022). Antioxidant enzymes and certain metabolites play important roles in adaptation and ultimate survival of plants under stress conditions. This study illustrated that antioxidant enzymes increased firstly and then decreased with the rising planting density and N addition. Wang et al. (2017) found that SOD of *C. camphora* seedlings decreased with the rising planting density. Dong et al. (2017); Chen et al. (2025); Hou et al. (2024), and Wang et al. (2023b) found that N supplementation could increase the activities of SOD, POD, and CAT to alleviate stress. The present study is consistent with the results of Wang et al. (2017); Dong et al. (2017); Chen et al. (2025); Hou et al. (2024), and Wang et al. (2023b). It illustrated that planting density and N addition could greatly influence the activity of antioxidant enzymes. It is mainly ascribed to dilution effect by high planting density and N addition. In addition, plants would increase the activity of antioxidant enzymes to further alleviate the harm, because elevated activity of antioxidative defense enzymes can serve as a better intrinsic defense tool to resist Mn-induced oxidative damage. Thus, plant tolerance would be enhanced by improving antioxidant defense ability through increasing planting density and N addition. However, when planting density and N supplying increased to a certain level, oxidative damage would increase due to limited resources by serious intraspecific competition and the imbalance of soil nutrients. Thus, the activity of antioxidant enzymes would decrease. Therefore, plants can effectively minimize Mn toxicity by improving antioxidative systems and scavenging ROS overproduction, which is an important mechanism for *X. strumarium* subsp*. sibiricum* to mitigate Mn stress.

The present study showed that MDA content and relative conductivity of *X. strumarium* subsp*. sibiricum* decreased firstly and then increased with the enhancing planting density and N application. The increase of MDA contents and loss of membrane integrity by high planting density and N application imply that high competition among individual plants would bring out the production of excessive ROS, resulting in an increase in lipid peroxidation. Thus, the plasma membrane was damaged and physiological activities related to membrane function would decrease under high planting density (84 individuals m^−2^) and N application (30 mmol L^−1^) and Mn stress. This is in line with the results of plant biomass, which showed that high planting density and N application decreased individual plant biomass under Mn stress.

### 4.3 The effects on Mn uptake

Planting density and N addition are important factors affecting heavy metal extraction capacity of plants, because they can change the environmental conditions that plants grow, which will influence heavy metal uptake and accumulation (Viana et al., 2021). This study revealed that Mn uptake by *X. strumarium* subsp*. sibiricum* increased and the dose per plant decreased with the rising planting density under Mn stress (**Figure 6**, **7**). Lu et al. (2010) and Hansi et al. (2014) showed that heavy metal concentrations of *Boehmeria nivea* decreased and heavy metal uptake increased as planting density increased under stress, whereas Jacobs et al. (2018) illustrated that concentrations of Cd and Zn in the plant of *Noccaea cadeulescens* increased under high planting density and stress. The present study is consistent with the results of Lu et al. (2010) and Hansi et al. (2014) and inconsistent with Jacobs et al. (2018). This is due to the dilution effect with high biomass with the rising planting density. Plant population biomass would enhance as planting density increased, individual plants sharing, and “competing” for heavy metals would happen under high heavy metal stress (Hansi et al., 2014). It leads to plant Mn concentration decrease as the planting density of *X. strumarium* subsp*. sibiricum* increases. Meanwhile, population biomass increases accompanied by rising planting density, which causes total Mn uptake to increase (Hansi et al., 2014). The discrepancy in plant heavy metal uptake between this study and Jacobs et al. (2018) under heavy metal stress may be due to being species-specific. As a hyperaccumulator plant, the roots of *Noccaea caerulescens* have strong tolerance to Cd and Zn and can enhance root proliferation to increase soil exploration under stress (Jacobs et al., 2018). More studies are needed to clarify this phenomenon. Meanwhile, this study showed that increasing planting density would increase total heavy metal uptake due to increased population biomass (**Figure 7**), whereas Mn uptake efficiency significantly decreased with rising planting density, which was indicated by a low plant Mn concentration (**Figure 6**). This suggested that appropriate planting density (e.g. 42 individuals m^−2^ for *X. strumarium* subsp*. sibiricum*) would benefit heavy metal uptake, because it can enhance phytoremediation efficiency by increasing plant production and influencing the bioavailability of heavy metals in soils (Liu et al., 2009; Viana et al., 2021; Zou et al., 2021).

The present study showed that Mn concentrations in individual plant of *X. strumarium* subsp*. sibiricum* decreased, whereas Mn uptake increased firstly and then decreased with the rising N application (**Figures 6**, **7**). The present result is in agreement with Jacobs et al. (2018). The main reason is that a dilution effect of accumulated metals within larger plants has happened and N might compete with Mn for root uptake. Although N application can enhance plant uptake heavy metal, N fertilization may compete partly with Mn, as found by Xie et al. (2009) in a rhizobox experiment. In this study, CO(NH_2_)_2_ was applied to the soil, where it can generate substantial amounts of ammonium (NH_4_^+^). NH_4_^+^ would have a stronger competition with cation and reduce metal uptake compared with nitrate (NO_3_^−^). This effect reinforces the hypothesis that NO_3_^−^ and NH_4_^+^ supply affects Mn uptake (Souza Junior and Monteiro, 2024). Furthermore, the overall negative effect of N fertilization on Mn concentration might also be partly explained by a lower root growth in N fertilized plants, which is in line with the results of the ratio of root to shoot (**Figure 1**). This result indicates that increasing planting density and N application might decrease the Mn uptake efficiency, although the biomass significantly increased. According to plant growth, physiological and biochemical characteristics, and Mn uptake efficiency, we thought that the treatment of planting density at 42 individuals m^−2^ and N addition at 5 mmol L^−1^ is an appropriate condition for the plant growth of *X. strumarium* subsp*. sibiricum*.

Heavy metal ions that arrive to the root system of plants would firstly deposit on the surface of roots, and then it would get into calyptrogen as apoplast. Next, it would enter into a vascular cylinder of the root and move upward to overground by transpiration. This study illustrated that BF of *X. strumarium* subsp*. sibiricum* decreased with the rising planting density and N application under Mn stress. Moreover, the TF of *X. strumarium* subsp*. sibiricum* increased with the rising planting density (**Figure 8**). It was reported that enhancing planting density decreased BF (Lu et al., 2010) and increased TF (Chen et al., 2022) under heavy metal stress. The present study is consistent with those of Lu et al. (2010) and Chen et al. (2022). The lower BF with higher planting density and N application may be due to the dilution effect. Dilution effect enhances plant biomass and decreases Mn uptake, which would result in low BF. Furthermore, the higher TF of *X. strumarium* subsp*. sibiricum* with higher planting density may ascribe to the competition for light. As plant growth increases with the rising of planting density, the level of competition for light, nutrition, and space among different individual plants would enhance. Then, plant water transpiration would increase, which would cause plants to uptake more heavy metal and allocate to different parts of the plant through the plastid flow pathway (Chen et al., 2022).

## 5 Conclusion

This study illustrated that both planting density and N application under Mn stress had great impacts on individual plant growth and physiological and biochemical characteristics, which appeared consistent. An appropriate planting density, such as the planting density of 42 individuals m^−^², together with 5 mmol/L N application, could promote the growth and Mn uptake of *X. strumarium* subsp*. sibiricum.* Mn stress could be mitigated by increasing the contents of osmotic adjustment substances, enhancing the activities of antioxidant enzymes, and reducing Mn concentrations in plants. Overall, this study elaborated the detoxification mechanism of cultivation measures of *X. strumarium* subsp*. sibiricum* from the perspective of their influences on individual plant competition, dilution effect, and physiological and biochemical characteristics, providing new insights for better understanding the enhancement of enrichment plant Mn extraction by planting density and N application. Moreover, the present study demonstrated in a pot experiment that managing planting density and N application are an efficient way to increase phytoextraction, as planting density and N addition affected Mn absorption and plant biomass production. More studies are needed to further reveal the tolerant mechanisms of plants under heavy metal stress with different planting density and N application.

## Data Availability

The original contributions presented in the study are included in the article/**Supplementary Material**. Further inquiries can be directed to the corresponding author.

## Supplementary material

Supplementary Table 1 Three-way ANOVAs of planting densities, nitrogen application, and Mn stress on root biomass, stem biomass, leaf biomass, fruit biomass, total biomass, and root–shoot ratio of *X. strumarium* subsp*. sibiricum*.

Supplementary Table 2 Three-way ANOVAs of planting densities, nitrogen application, and Mn stress on chlorophyll a, chlorophyll b, chlorophyll a/b, and carotenoids of *X. strumarium* subsp*. sibiricum*.

Supplementary Table 3 Three-way ANOVAs of planting densities, nitrogen application, and Mn stress on soluble sugar, soluble protein, and free proline of *X. strumarium* subsp*. sibiricum*.

Supplementary Table 4 Three-way ANOVAs of planting densities, nitrogen application, and Mn stress on superoxide dismutase (SOD), peroxidase (POD), and catalase (CAT) of *X. strumarium* subsp*. sibiricum*.

Supplementary Table 5 Three-way ANOVAs of planting densities, nitrogen application, and Mn stress on MDA content and relative conductivity of *X. strumarium* subsp*. sibiricum*.

Supplementary Table 6 Three-way ANOVAs of planting densities, nitrogen application, and Mn stress on root Mn concentration, stem Mn concentration, leaf Mn concentration, fruit Mn concentration, and total Mn concentration of *X. strumarium* subsp*. sibiricum*.

Supplementary Table 7 Three-way ANOVAs of planting densities, nitrogen application, and Mn stress on root Mn uptake, stem Mn uptake, leaf Mn uptake, fruit Mn uptake, and total Mn uptake of *X. strumarium* subsp*. sibiricum*.

Supplementary Table 8 Three-way ANOVAs of planting densities, nitrogen application, and Mn stress on the shoot bioconcentration factor, root bioconcentration factor, individual plant bioconcentration factor, and translocation factor of *X. strumarium* subsp*. sibiricum*.
