# Supplementary material for: Effects of planting density and nitrogen application on the growth of Xanthium strumarium subsp. sibiricum under manganese stress
Source: Front Plant Sci. 2026 Jan 20;16:1704841. doi: 10.3389/fpls.2025.1704841 (PMC12864105; doi:10.3389/fpls.2025.1704841)
Supplement: Supplementary Table 1 — Three-way ANOVAs of planting densities, nitrogen application, and Mn stress on root biomass, stem biomass, leaf biomass, fruit biomass, total biomass, and root–shoot ratio of X. strumarium subsp. sibiricum. [file DataSheet1.doc]

**Supplementary data**

**Table S1** Three-way ANOVA analysis of planting densities, nitrogen application, and Mn stress on root biomass, stem biomass, leaf biomass, fruit biomass, total biomass and root-shoot ratio of *X. strumarium* subsp*. sibiricum*.

| Factors | Root biomass | Stem biomass | Leaf biomass | Fruit biomass | Total biomass | Root shoot ratio |
| --- | --- | --- | --- | --- | --- | --- |
| Mn | 862.253** | 835.848** | 2263.453** | 5496.850** | 5717.059** | 226.591** |
| PD | 62.812** | 94.653** | 189.534** | 479.905** | 503.294** | 26.155** |
| N | 738.322** | 193.965** | 461.586** | 560.002** | 1143.167** | 37.724** |
| Mn×PD | 40.600** | 81.527** | 266.918** | 386.212** | 466.468** | 33.139** |
| Mn×N | 19.527** | 45.324** | 138.018** | 410.050** | 225.332** | 50.939** |
| PD×N | 60.065** | 28.530** | 45.647** | 158.674** | 154.697** | 11.862** |
| Mn×PD×N | 52.417** | 33.915** | 56.073** | 112.578** | 162.379** | 2.810* |

**Table S2** Three-way ANOVA analysis of planting densities, nitrogen application, and Mn stress on chlorophyll a, chlorophyll b, chlorophyll a/b, and carotenoids of *X. strumarium* subsp*. sibiricum*.

| Factors | Chlorophyll a | Chlorophyll b | Chlorophyll a/b | Carotenoids |
| --- | --- | --- | --- | --- |
| Mn | 3021.605** | 294.576** | 7.281* | 382.652** |
| PD | 367.673** | 30.725** | 2.381ns | 19.007** |
| N | 830.346** | 122.999** | 2.163ns | 35.951** |
| Mn×PD | 729.793** | 32.457** | 13.159** | 35.435** |
| Mn×N | 350.477** | 2.913ns | 22.274** | 19.636** |
| PD×N | 104.188** | 10.554** | 3.626* | 5.600** |
| Mn×PD×N | 241.897** | 7.008** | 11.071** | 9.312** |

**Table S3** Three-way ANOVA analysis of planting densities, nitrogen application, and Mn stress on soluble sugar, soluble protein, and free proline of *X. strumarium* subsp*. sibiricum*.

| Factors | soluble sugar | soluble protein | free proline |
| --- | --- | --- | --- |
| Mn | 271.775** | 384.955** | 439.099** |
| PD | 72.666** | 43.833** | 43.510** |
| N | 247.385** | 131.052** | 123.482** |
| Mn×PD | 119.166** | 46.752** | 53.204** |
| Mn×N | 12.840** | 36.956** | 3.116ns |
| PD×N | 21.809** | 6.101** | 14.271** |
| Mn×PD×N | 8.560** | 9.977** | 10.865** |

**Table S4** Three-way ANOVA analysis of planting densities, nitrogen application, and Mn stress on superoxide dismutase (SOD), peroxidase (POD), and catalase (CAT) of *X. strumarium* subsp*. sibiricum*.

| Factors | SOD | POD | CAT |
| --- | --- | --- | --- |
| Mn | 5727.169** | 1166.921** | 3501.576** |
| PD | 396.900** | 274.071** | 289.545** |
| N | 5449.866** | 971.119** | 1452.858** |
| Mn×PD | 146.297** | 118.281** | 487.087** |
| Mn×N | 342.442** | 49.773** | 61.714** |
| PD×N | 321.327** | 65.890** | 146.849** |
| Mn×PD×N | 287.290** | 62.908** | 62.863** |

**Table S5** Three-way ANOVA analysis of planting densities, nitrogen application, and Mn stress on MDA content and relative conductivity of *X. strumarium* subsp*. sibiricum*.

| Factors | MDA | Relative conductivity |
| --- | --- | --- |
| Mn | 3340.789** | 4892.713** |
| PD | 171.242** | 158.885** |
| N | 414.733** | 753.235** |
| Mn×PD | 177.778** | 174.880** |
| Mn×N | 80.874** | 177.356** |
| PD×N | 74.537** | 46.626** |
| Mn×PD×N | 39.332** | 97.151** |

**Table S6** Three-way ANOVA analysis of planting densities, nitrogen application, and Mn stress on root Mn concentration, stem Mn concentration, leaf Mn concentration, fruit Mn concentration, and total Mn concentration of *X. strumarium* subsp*. sibiricum*.

| Factors | Root Mn  concentration | Stem Mn  concentration | Leaf Mn  concentration | Fruit Mn  concentration | Total Mn  concentration |
| --- | --- | --- | --- | --- | --- |
| Mn | 294015.212** | 2781791.665** | 8478204.539** | 136571.781** | 24573.788** |
| PD | 6686.212** | 14148.602** | 17052.723** | 2130.117** | 54.369** |
| N | 1423.822** | 8403.042** | 6459.910** | 1307.927** | 193.447** |
| Mn×PD | 2892.545** | 11783.452** | 11514.642** | 1258.806** | 37.203** |
| Mn×N | 1236.249** | 4832.859** | 3771.927** | 780.382** | 171.144** |
| PD×N | 186.248** | 659.837** | 842.247** | 27.091** | 0.691ns |
| Mn×PD×N | 184.503** | 709.546** | 457.826** | 9.304** | 0.398ns |

**Table S7** Three-way ANOVA analysis of planting densities, nitrogen application, and Mn stress on root Mn uptake, stem Mn uptake, leaf Mn uptake, fruit Mn uptake, and total Mn uptake of *X. strumarium* subsp*. sibiricum*.

| Factors | Root Mn  uptake | Stem Mn  uptake | Leaf Mn  uptake | Fruit Mn  uptake | Total Mn  uptake |
| --- | --- | --- | --- | --- | --- |
| Mn | 21387.149** | 5949.014** | 7917.943** | 1969.268** | 10185.978** |
| PD | 7081.131** | 1886.788** | 2087.250** | 1221.885** | 2814.351** |
| N | 705.977** | 206.495** | 67.013** | 175.273** | 128.208** |
| Mn×PD | 5084.930** | 1398.708** | 1893.681** | 508.060** | 2427.941** |
| Mn×N | 467.780** | 168.288** | 56.598** | 158.773** | 105.122** |
| PD×N | 197.652** | 61.211** | 21.355** | 42.440** | 38.664** |
| Mn×PD×N | 145.458** | 49.602** | 18.205** | 36.124** | 32.266** |

**Table S8** Three-way ANOVA analysis of planting densities, nitrogen application, and Mn stress on the shoot bioconcentration factor, root bioconcentration factor, individual plant bioconcentration factor, and translocation factor of *X. strumarium* subsp*. sibiricum*.

| Factors | Shoot bioconcentration factor | Root bioconcentration factor | Individual plant bioconcentration factor | translocation factor |
| --- | --- | --- | --- | --- |
| Mn | 21340.948** | 65427.469** | 4457.028** | 8743.493** |
| PD | 1394.060** | 7651.188** | 323.970** | 320.545** |
| N | 1689.897** | 123.814** | 162.283** | 58.352** |
| Mn×PD | 1134.542** | 6214.914** | 233.596** | 54.237** |
| Mn×N | 578.502** | 52.799** | 44.866** | 15.576** |
| PD×N | 128.287** | 27.264** | 10.955** | 7.475** |
| Mn×PD×N | 132.368** | 26.604** | 9.412** | 11.702** |
